# Study of Methionine Choline Deficient Diet-Induced Steatosis in Mice Using Endogenous Fluorescence Spectroscopy

**DOI:** 10.3390/molecules24173150

**Published:** 2019-08-29

**Authors:** Alma Valor, Eduardo J. Arista Romeu, Galileo Escobedo, Adriana Campos-Espinosa, Ivette Irais Romero-Bello, Javier Moreno-González, Diego A. Fabila Bustos, Suren Stolik, Jose Manuel de la Rosa Vázquez, Carolina Guzmán

**Affiliations:** 1 Laboratorio de Biofotónica, ESIME Zac, Instituto Politécnico Nacional, Ciudad de Mexico 07738, Mexico; 2 Laboratorio de Proteómica, Dirección de Investigación, Hospital General de Mexico “Dr. Eduardo Liceaga”, Ciudad de Mexico 06720, Mexico; 3 Laboratorio de Hígado, Páncreas y Motilidad, Unidad de Medicina Experimental, Facultad de Medicina, Universidad Nacional Autónoma de Mexico/Hospital General de Mexico “Dr. Eduardo Liceaga”, Ciudad de Mexico 06720, Mexico; 4 Laboratorio de Espectroscopia, UPIIH, Instituto Politécnico Nacional, Ciudad del Conocimiento y la Cultura, San Agustín Tlaxiaca 42162, Mexico

**Keywords:** endogenous fluorescence spectroscopy, liver steatosis, multi-variate analysis

## Abstract

Non-alcoholic fatty liver disease is a highly prevalent condition worldwide that increases the risk to develop liver fibrosis, cirrhosis, and hepatocellular carcinoma. Thus, it is imperative to develop novel diagnostic tools that together with liver biopsy help to differentiate mild and advanced degrees of steatosis. Ex-vivo liver samples were collected from mice fed a methionine-choline deficient diet for two or eight weeks, and from a control group. The degree of hepatic steatosis was histologically evaluated, and fat content was assessed by Oil-Red O staining. On the other hand, fluorescence spectroscopy was used for the assessment of the steatosis progression. Fluorescence spectra were recorded at excitation wavelengths of 330, 365, 385, 405, and 415 nm by establishing surface contact of the fiber optic probe with the liver specimens. A multi-variate statistical approach based on principal component analysis followed by quadratic discriminant analysis was applied to spectral data to obtain classifiers able to distinguish mild and moderate stages of steatosis at the different excitation wavelengths. Receiver Operating Characteristic (ROC) curves were computed to compare classifier’s performances for each one of the five excitation wavelengths and steatosis stages. Optimal sensitivity and specificity were calculated from the corresponding ROC curves using the Youden index. Intensity in the endogenous fluorescence spectra at the given wavelengths progressively increased according to the time of exposure to diet. The area under the curve of the spectra was able to discriminate control liver samples from those with steatosis and differentiate among the time of exposure to the diet for most of the used excitation wavelengths. High specificities and sensitivities were obtained for every case; however, fluorescence spectra obtained by exciting with 405 nm yielded the best results distinguishing between the mentioned classes with a total classification error of 1.5% and optimal sensitivities and specificities better than 98.6% and 99.3%, respectively.

## 1. Introduction

The anomalous accumulation of lipids in the liver of patients with negligible alcohol consumption and seronegative to hepatitis C virus infection (HCV) is called non-alcoholic fatty liver disease (NAFLD) and is characterized by a broad spectrum of hepatic histopathological changes [1]. NAFLD can potentially progress from the accumulation of lipids generally found in simple steatosis to nonalcoholic steatohepatitis (NASH), eventually followed by cirrhosis, hepatocellular carcinoma (HCC), and liver failure [2]. For this reason, it is of great importance to carry out a precise evaluation of simple steatosis in order to identify patients at higher risk of such evolution [3].

The gold standard technique for rating the degree of NAFLD is the liver biopsy [4,5]. Numerous studies have consistently reported significant intra- and inter-observer variability in the assessment of intrahepatic lipid accumulation, especially in mild-to-moderate and moderate-to-advanced steatosis degrees by means of a liver biopsy [3,4,5,6,7,8,9,10]. Unfortunately, such a marked variability may result in an underestimation of the degree of steatosis and an inadequate diagnosis of the disease, which could consequently lead to poor medical treatment and follow-up for patients [6,11]. Therefore, it is imperative to establish techniques complementary to liver biopsy in order to more precisely grade hepatic steatosis in patients at risk of developing chronic liver disease.

Recently, diagnostic techniques based on the interaction of light with biological materials have been developed. These techniques have been called optical biopsy or optical diagnosis by several authors. They use the light absorption and scattering to quantify the biochemical composition and morphological characteristics of tissue in a noninvasive or minimally invasive manner [12]. Some of these existing optical techniques are based on fluorescence which is produced by a variety of endogenous fluorophores such as NAD(H), NAD, flavins, porphyrins, and collagen, among others [13]. The amount and distribution of these fluorophores can be modified by pathological conditions producing a modification of the fluorescence emission of the tissue. Several studies in different tissues have been carried out to evaluate the feasibility of fluorescence spectroscopy as a diagnostic tool, particularly in tumor cells [14,15].

Endogenous fluorescence spectroscopy (EFS) has recently been used to study metabolic changes induced by the accumulation of fat in the liver [16]. In this direction, Croce et al. [17] established that endogenous fluorescence spectra obtained in-vivo and ex-vivo after excitation at 366 nm of rat liver samples with micro and macro vesicular steatosis significantly differ from those obtained from control liver samples without lipid deposits. A similar result has been reported in a different study of NAFLD also performed in an animal model [18] using an excitation light of 532 nm. However, there is still insufficient experimental evidence regarding the use of endogenous hepatic fluorescence spectra as a diagnostic tool to recognize mild and moderate stages of NAFLD.

The central purpose of this work was to examine whether the liver tissue has specific endogenous fluorescence patterns depending on the degree of steatosis, and assessing the feasibility of grading liver steatosis by means of EFS as a complementary tool to liver biopsy using an animal model of NAFLD.

## 2. Results

### 2.1. Morphometric Determination of Lipid Content

After the morphometric analysis [19], the mean hepatic fat content did not show significant differences with a 0.05 significance level between mice fed a methionine-choline deficient (MCD) diet for two (MCD2w) or eight weeks (MCD8w). On the contrary, liver fat content of control group (MCC) was clearly different from both MCD2w and MCD8w groups (MCC = 0.16 ± 0.35%, MCD2w = 6.73 ± 5.78%, MCD8w = 10.99 ± 6.83%; *p* < 0.05). In other words, morphometric determination is able to distinguish between control group and the liver groups with induced steatosis but fails to show significant differences between the two studied steatosis degrees.

### 2.2. Fluorescence Spectra Characterization

**Figure 1:**
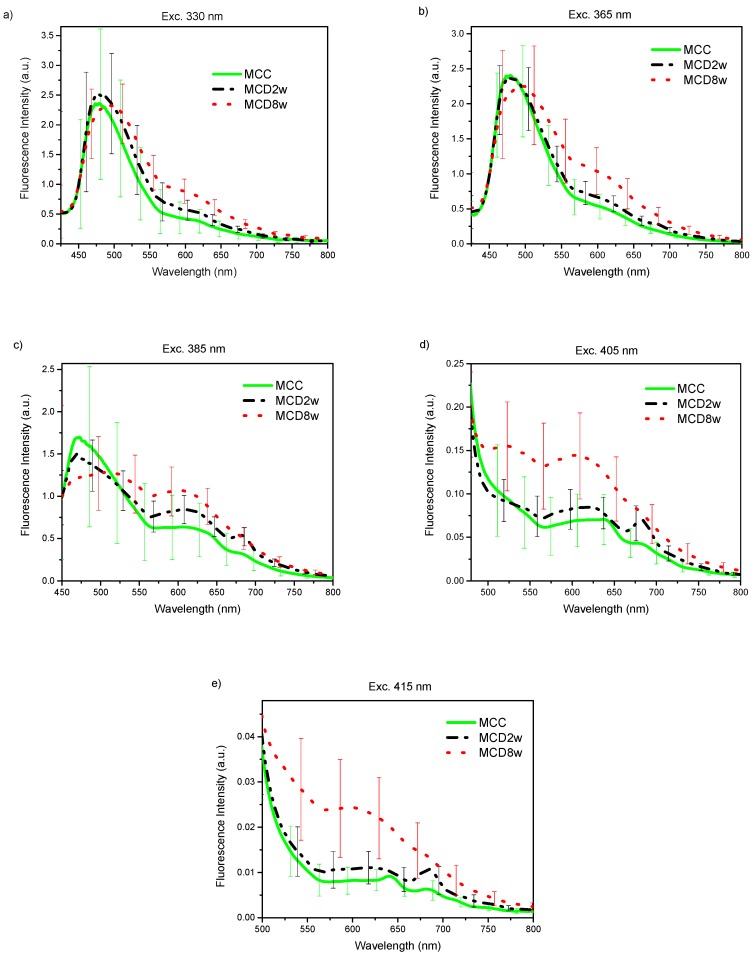
Endogenous fluorescence spectra from control (MCC), and steatosis (MCD2w, and MCD8w) groups, after the spectral sensitivity correction, at excitation wavelengths of (**a**) 330, (**b**) 365 nm, (**c**) 385 nm, (**d**) 405 nm, and (**e**) 415 nm. The spectra are presented as mean ± standard deviation of all gathered spectral observations.

The acquired endogenous fluorescence spectra using excitation wavelengths of 330, 365, 385, 405, and 415 nm are shown in Figure 1a–e. The plotted curves represent the mean spectrum of all spectral observations gathered for each excitation wavelength on liver samples corresponding to each one of the groups of animals as classified by their exposure time to a specific diet [20]. The presented spectra have been normalized at an arbitrary wavelength of 450 nm. The error bars in the graphics correspond to the standard deviation for all observed spectra of one group. In all cases, no statistical pre-processing of the spectra has been carried out, and all measurements are included.

In general, regardless the used excitation wavelength, exposure time of animals to the MCD diet progressively increases fluorescence intensity in the liver, especially in the range from 550 nm upward. This increase is more dramatic for larger excitation wavelengths 385, 405, and 415 nm.

### 2.3. Analysis of Fluorescence Spectra and the Determined Lipid Content

**Figure 2:**
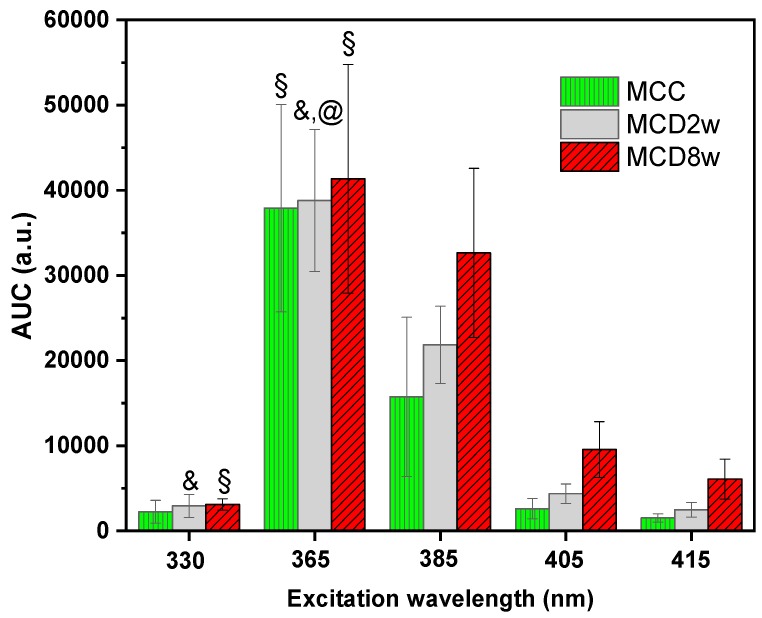
Area under the curve (AUC) of the spectra measured on liver samples of the three different groups MCC, MCD2w and MCD8w—at different excitation wavelengths indicated in the horizontal axis. The bar heights correspond to the mean AUC value for one group and the error-bars stand for the corresponding standard deviation. The symbols over some of the bars indicate that the null hypothesis that the given group is significantly different from some other group could be rejected at *p* < 0.05 (Tukey test): @—versus MCC, **§**—versus MCD2w, **&**—versus MCD8w.

For the analysis of the fluorescence measurements and its correlation with the liver lipid content, a global characteristic of the spectra, the area under the curve (AUC), was used. In Figure 2, a bar graph of the AUC of the spectra observed for the three liver groups MCC, MCD2w, and MCD8w at different excitation wavelengths are shown. The spectral integration intervals are 425–800 nm, 425–800 nm, 440–800 nm, 490–800 nm, and 500–800 nm, respectively, for the excitation wavelengths of 330, 365, 385, 405, and 415 nm. Note that the lower limit of the integration intervals increases with the increment of the excitation wavelength due to the increasing proximity of the excitation band to that lower limit. However, this change in the integration interval does not affect the comparison between the AUC values calculated for spectra measured on livers of the three groups at the same excitation wavelength.

To evaluate the capability of endogenous fluorescence spectroscopy to differentiate between different stages of steatosis, an ANOVA analysis was carried out to analyze the AUC mean values. The AUC mean values of the three groups are statistically different at excitation wavelengths of 385, 405, and 415 nm as confirmed by Tukey procedure at *p*-value of *p <* 0.05. However, for the excitation light of 365 nm, only the difference between the AUC corresponding to MCC and MCD8w groups was significant at the *p <* 0.05 level. Meanwhile, for the excitation light of 330 nm, only the differences between the means of the MCC and MCD8w groups and between the means of the MCC and MCD2w groups were significant at *p <* 0.05. In Figure 2, only the cases where the null hypothesis (that the given group is significantly different from the group to which it is compared) could be rejected are highlighted, using for that the following symbols: “@”—the given group compared versus MCC, “§”—versus MCD2w, and “&”—versus MCD8w. The absence of symbol corresponds to cases where AUC constitutes an adequate differentiation parameter between the groups.

To evaluate the correlation of the mean AUC values of the fluorescence spectra for each specimen (mouse) with the lipid content assessed by morphometry, the corresponding Spearman correlation coefficients were calculated; the results are presented in Table 1. Diminished correlation coefficients can be observed for the excitation wavelengths of 330 and 365 nm. The coefficient is not statistically significant, especially for the latter. These results might be related to the dissimilar way in which the fluorescence spectra manifest in the region below 515 nm when excitation wavelengths of 330 and 365 nm are used [21]. In general, the obtained correlation coefficients for every excitation wavelength are lower than those previously reported for the diffuse reflectance spectra [19].

Nonetheless, the AUC for fluorescence spectra with different excitation wavelengths used in the present investigation show a similar behavior, increasing its value with the time of exposure to the MCD diet. This fact is reflected in the Spearman correlation coefficient, as seen in Table 1, for the case of fluorescence excitation with wavelengths equal or larger than 385 nm. The correlation coefficients indicate a monotonic (Spearman) association between the spectral AUC and the liver lipid content with a significance level of 0.05. It is therefore appropriate to conclude that the AUC is an adequate variable to differentiate between the two analyzed stages of liver steatosis only for the higher excitation wavelengths: 385, 405, and 415 nm.

### 2.4. Principal Component Analysis (PCA) and Discriminant Analysis (DA)

The first six principal component (PC) values were computed and selected for a supervised classification scheme. These six PCs accounted for 99.44% of the variance for the 330 nm excitation wavelength, for 99.98% of the variance in the case of the 365 nm excitation; for 99.43% of the variance at 385 nm excitation, 98.23% at 405 nm, and for 99.08% of the variance at 415 nm.

For the linear discriminant analysis (LDA), the equality test of the group covariance matrices for each data set failed at a significance level of 0.05 (likelihood-ratio test is not shown). In such a case, the quadratic variant of the discriminant analysis is recommended for which the boundaries between the classes are second order expressions that typically improve the performance of the classifier. Quadratic discriminant analysis (QDA) was implemented, and its confusion matrixes (actual versus predicted values) were calculated and are shown in Table 2 for all excitation lights as values that appear before the comma in the table’s cells.

**Figure 3:**
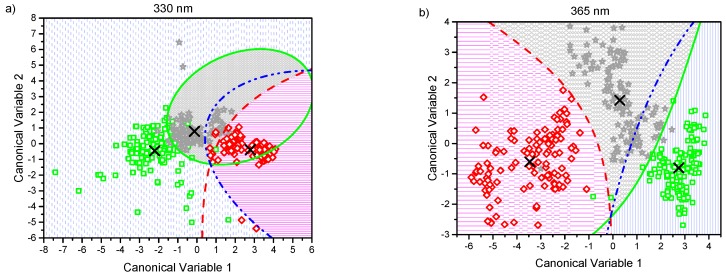

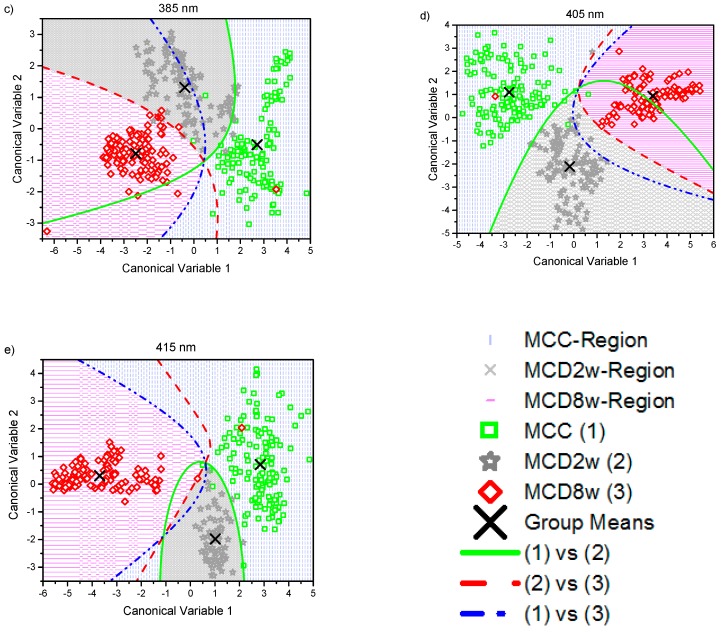
Canonical score plots obtained by the quadratic discriminant analysis (QDA) applied to the six firsts principal component (PC) values obtained from all observed spectra normalized at 600 nm using excitation lights of: (**a**) 330 nm, (**b**) 365 nm, (**c**) 385 nm, (**d**) 405 nm, and (**e**) 415 nm. Distribution of every single collected measurement are represented for every group in their corresponding regions: MCC (green squares in blue pattern), MCD2w (gray stars in gray pattern), and MCD8w (red diamonds in pink pattern). The mean value of each group is represented by an “×.” The curved lines mark the boundaries between regions. The inset in the lower right corner serves as a common legend to all the graphs.

The canonical score plots for the DA are shown in Figure 3a–e. There, a point representing the mean value of the canonical variables for each classification group (corresponding to MCC, MCD2w, and MCD8w) has been marked with the symbol “×.” Every single collected measurement is also represented for every group in their corresponding regions as determined by the classification scheme. The canonical variables corresponding to those measurements taken from MCC group are displayed as green squares in a blue vertical pattern region. MCD2w points are marked as grey stars in a grey cross pattern, and MCD8w points are shown as red diamonds in a pink horizontal pattern. In the same figure, the boundaries between regions as resolved by the model are plotted as curved lines. From the simple observation of Figure 3, the discrimination between all groups is possible.

Table 3 summarizes the classification and cross validation error rates for the QDA models for the different excitation wavelengths. To obtain the error rate for each group, the diagonal values of the respective confusion matrixes that reflect the correct group classification rate are subtracted from 100% corresponding to the whole membership of the group. Error values in the column named “Total” account for the percent of misclassifications relative to the actual global number of observations for each excitation wavelength, adjusted for membership differences between the groups.

In Table 3, cross-validation error rates for all excitations lights were close enough to those of the respective MCC, MCD2w, and MCD8w groups to validate the used classification model for each set of data. Receiver Operating Curves (ROC) describing the capacity of correctly assigning any of the measurements to its group were obtained for the QDA model (Table 4) for the following cases: control (MCC) vs simple steatosis (MCD2w and MCD8w), MCD2w vs (MCC and MCD8w), and MCD8w vs (MCD2w and MCC) for all excitation wavelengths. It can be noted that the mean AUC was larger than 0.98, which is close to the ideal value of one.

The Youden *J*-statistic for each ROC curve were calculated and averaged for each excitation wavelength, its maximum value, the optimal cut-off point for a ROC curve is reported in Table 4 for each class in every excitation wavelength. The obtained mean sensitivities and specificities were better than 95.6% and 93.3%, respectively, and Youden index values were located closely to each other.

The large values of the AUC for ROC curves as well as the high sensitivities and specificities obtained and reported in Table 4 talk about the good performance of the used classification model and point to the value of using autofluorescence as a tool for the identification of progression from mild steatosis to moderate steatosis and to discriminate both liver steatosis stages from healthy livers and its capacity of correctly assigning any of the measurements to its correct group.

## 3. Discussion

### 3.1. Spectral Characteristics

For all the excitation wavelengths, it was observed that, as NAFLD progresses, the fluorescence intensity increases in the wavelength range above 500 nm (Figure 1). A plausible explanation for this fact can be found in the increment of hepatic tissue diffuse reflectance in the same wavelength interval [19] that causes an increment in the mean free path of photons that, in turn, increases the possibility of light being absorbed to give rise to a greater number of fluorescence events. In this same tenor, the most evident increase in the fluorescence intensity with disease progression for the excitation wavelengths of 385, 405, and 415 nm can be explained by a greater value of the light mean-penetration-depth in liver tissue for these wavelengths than in the case of excitation with wavelengths of 330 and 365 nm [22,23].

The graphs of the spectra (Figure 1) are consistent with the reported histological information, where an increase in tissue heterogeneity along with the fat content is observed. These heterogeneities, associated to the advance of fatty liver disease, should be accountable for the observed growth in the standard deviation of the fluorescence spectra with the increase in the time of exposure to the MCD diet.

The variation in the shape of the fluorescence spectra is conditioned by the structural and compositional changes that the hepatic tissues undergo with the progression of NAFLD. Based on the absorption and emission spectra of the main fluorophores present in the liver tissue [14] and the wavelength of the excitation light sources used to induce fluorescence, a qualitative analysis of the variations in the spectra of Figure 1 can be made.

With the excitation light of 330 nm (Figure 1a), a fluorescence peak with a maximum around 475 nm and extending beyond 550 nm is observed for the MCC group that widens and moves towards the longer wavelengths with the progression of the NAFLD. This peak can respond to the fluorophores present in the liver as vitamin A with an absorption band whose maximum is at 328 nm [24] and arachidonic acid [17,25,26], and fundamentally pyridine nucleotides NAD(P)H (in bound or free state) and flavins (FAD) [27,28]. The account for the peak redshift upon steatosis advance could be found in the change of the metabolic redox parameters, as is the case of the increase in the NAD(P)H_bound/free_ ratio, as proposed by Croce et al. [17], and the increase in the redox ratio [17,27] with the decrease of metabolic activity that accompanies the progression of the disease. It is worth noting that, after 525 nm, the fluorescence intensity steadily increases with the exposure time to the MCD diet. In this wavelength range, the main liver endogenous fluorophores are lipopigments, lipofuscin, and porphyrins [25,27].

With excitation wavelength of 365 nm (Figure 1b), the graph is very similar to the previous one, but the redshift of the first peak is more evident probably because at 365 nm excitation wavelengths FAD have an absorption maximum, which makes the fluorescence coming from these compounds, with emission maximum at 520 nm, more intense. Again, after 515 nm, the fluorescence intensity increases with time of the MCD diet.

At 385 nm excitation wavelength (Figure 1c), the ample peak observed in the two previous cases is no longer so dominant because, at this wavelength, NAD(P)H, especially that in free state, are excited only marginally. The same is valid for vitamin A. However, the other fluorophores such as flavins, lipopigments, [29] and porphyrins [14] are excited. The initial peak changes shape by a more extensive contribution of lipopigments and flavins, to which corresponds a peak at 540 nm. The redshift is even more dramatic at this excitation wavelength as a result of the flavins being cofactors that participate in cellular redox processes and to the increased contribution of lipopigments with the MCD exposure progression as has been discussed in [17]. A secondary peak appears at 620 nm with the contributions of lipofuscin [30] and porphyrins. Also, the peak of secondary emission at 680 nm of porphyrins begins to become visible. The growth of the second fluorescence band with exposure time to MCD diet (see Figure 1c) can be ascribed to the growing presence of lipofuscin in NAFLD, as has been recently reported [31], and the role of cytochromes, whose prosthetic group is a porphyrin, in the ROS production during the steatosis progression. The minimum between the two main fluorescence bands corresponds to the minimum observed in the diffuse reflectance spectra analyzed in Reference [19] that responds to a maximum in the optical absorption properties by the tissues. Throughout the spectral range above 520 nm, the intensity of the fluorescence increases with time of the MCD diet.

With excitation light of 405 nm (Figure 1d), vitamin A and NAD(P)H are no longer excited, while that of porphyrins increase. Flavins, lipopigments, and lipofuscin are still excited. This excitation behavior together with the structural and metabolic processes taking place in the liver exposed to MCD diet explain the observed spectra of Figure 1d. The reduced contribution of the unexcited species can explain the general intensity decrease of the first fluorescence band, which remains due to the increasing presence of flavins and lipopigments with the advance of the MCD diet and the consequent steatosis. The relative contribution of the broad band at 620 nm, for which lipofuscin and porphyrin should be responsible, increases and also that of the secondary emission peak at 680 nm of the porphyrins. The latter is no longer discernible for the MCD8w spectrum, probably due to the superposition of the wide and intense band of 620 nm. Note that, throughout the observed spectral range, the intensity of the fluorescence generally increases with time of the MCD diet as a consequence of the metabolic and composition changes taking place in the liver.

For the 415 nm excitation wavelength (Figure 1e), the graph is very similar to the previous one (405 nm), with the main distinction that the first fluorescence band is less resolved probably due to the null excitation of NADH and vitamin A due to the excitation wavelength used. Thus, the wide peak at 620 nm to which lipofuscin and porphyrin contribute is the predominant one, and the secondary emission peak at 680 nm of the porphyrins is also visible. As in the previous case, in the observed spectral range, the fluorescence intensity increases with time of the MCD diet.

The measured fluorescence spectra can be viewed as a mixture of fluorescence emissions produced by all the liver fluorescent compounds present in every stage. With UV light excitation, as the fatty content in the liver rises, the fluorescence of the flavins and lipofuscin-like lipopigments also rises while the fluorescence of NADH, vitamin A and proteins diminish giving rise to a band redshift in Figure 1a,b under 525 nm. A more detailed study is needed in order to make quantitative estimations about the proportions of these fluorophores.

On the other hand, above 525 nm, for every excitation wavelength, the fluorescence intensity increases with the growth of liver fat content, mainly due to the increasing presence of porphyrins, lipofuscin, and lipopigments. This finding points to the possibility of making this intensity growth trend of the fluorescence band above 525 nm an optical marker for the detection and gradation of fatty liver disease.

### 3.2. Statisctics

The results obtained from of the ANOVA followed by Tukey tests for the AUC presented in Figure 2, shows that AUC have a clear increment especially for excitation wavelengths at 385, 405, and 415 nm, and in fact a statistical difference at *p* < 0.05 was obtained between different stages of steatosis. In contrast, for excitation wavelengths of 330 and 365 nm the obtained AUC show statistical difference at *p* < 0.05 only for some cases. The correlation coefficients for the comparison between fluorescence measurements and lipid quantification from morphometry of Table 1 for the 330 and 365 nm excitation lights are in correspondence with the fluorescence spectra in Figure 1a,b where, for the interval below 515 nm, the spectra behave differently. However, the large specific weight corresponding to the area under the wide band that appears below 520 nm in these two groups of spectra probably defines the impossibility of correctly correlating the fluorescence with the liver fat content, despite the fact that after 520 nm, the difference between spectra of each group is apparent.

For the case of fluorescence excited with wavelengths of 385, 405, and 415 nm, for which the first fluorescence band has less weight in the spectrum, the area under the curve adequately correlates with the fat content, and the groups, formed according to the fed diet, have fluorescence spectra that are statistically different judging by their AUC. It is worth noting that the diminished correlation coefficients of the area under the curve of fluorescence spectra versus lipid content compared to the correlation coefficients for the diffuse reflectance spectra reported in Reference [19] can be explained as a consequence of the band character of the fluorescence emission spectra, whose significance is diluted when calculating the area under the curve over the entire wavelength measurement interval.

**Figure 4:**
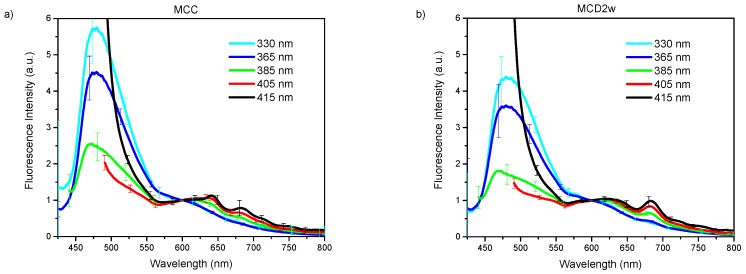

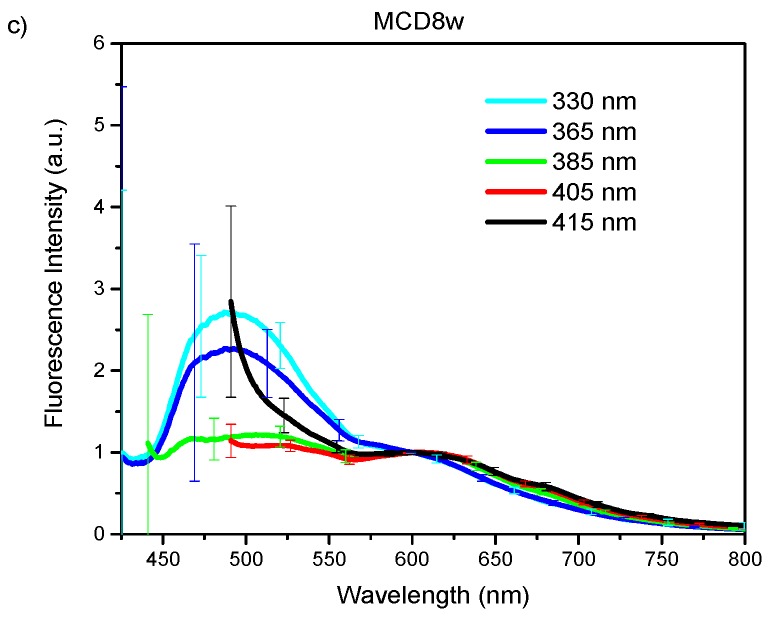
Group mean spectra with spectral sensitivity correction and normalization at 600 nm, used excitation lights are indicated in the legend, bars indicate the standard deviation: (**a**) MCC group; (**b**) MCD2w group; (**c**) MCD8w group.

As several excitations lights with different optical powers were used, to compare the fluorescence spectra obtained from them, the spectra were normalized at an arbitrarily selected wavelength of 600 nm (Figure 4). In this way, it can be visualized how fluorescence from a given dietary group changes as the excitation wavelength increases. The increase of the intensity in the spectral range above 600 nm with the excitation wavelength growth is apparent, owing to the excitation of porphyrins and lipofuscin, as it has been already discussed. Notable is the variation of the standard deviation that diminishes from 330 nm up to the 405 nm excitation light starting to increase with the 415 nm excitation. Also, the emission bands of some fluorophores as the porphyrins are clearly expressed. The information in this figure is the input to the classification algorithm we used.

The application of the PCA to the normalized and averaged spectra of different groups—distributed according to the diet fed to the animals and followed by the supervised classification and cross validation by QDA—showed a classification success higher than 87% in all cases, as can be seen in Table 3. The worst results in the classification are obtained for the lower excitation wavelengths (330 and 365 nm), presumably due to the contribution of the fluorescence band with wavelengths less than 520 nm, in which the differences between the three measured classes are not so evident. The best classification is obtained by spectral measurements with higher excitation wavelengths—405 and 415 nm, for which the band mentioned above does not appear. The classification errors of Table 3 confirm those results.

The effectiveness of the proposed classification scheme to differentiate pairwise between different grades of steatosis: control (MCC) versus mild steatosis (MCD2w), control vs moderate steatosis (MCD8w), and mild vs moderate can be also evaluated by observing the score plots in Figure 3, where the plotted classification boundaries between the groups indicate the success of the performed classification.

Average sensitivities of 95.6%, 97.9%, 98.0%, 99.0%, and 98.8% with specificities of 93.3%, 98.4%, 97.5%, 99.4%, and 98.1% were obtained, respectively, for the five excitation wavelengths. The best classification results correspond to the excitation light of 405 nm, with sensitivities of 98.6%, 99.2%, and 99.2% for the pairs control (MCC) vs steatosis (MCD2w & MCD8w), mild steatosis vs control plus moderate steatosis, and moderate steatosis vs control plus mild steatosis, respectively, and corresponding specificities of 99.6%, 99.3%, and 99.3% (see Table 4). These results are consistent with the measured fluorescence spectra shown in Figure 1d, where the intensity differences between the group mean spectra, in the interval above 600 nm, are larger than the respective group standard deviations.

In this work, we developed several tests to evaluate the capability of EFS as an assessment tool of liver steatosis in its early stages, starting with the use of the AUC of the measured spectra as a classification parameter. It was proven that the AUC is not a good enough classifier, especially for excitation wavelengths of 330 and 365 nm. However, the application of the proposed multivariate statistical analysis allowed us to develop a robust and more sophisticated scheme of classification that yields high sensitivities and specificities. The application of PCA enabled the use of all recorded fluorescence spectra with a minimum loss of information. PCA was followed by QDA, which produces a non-linear boundary model between classes (Figure 3), overcoming the shortcomings of linear classification models. This allowed us to generate a better algorithm contrasting with schemes where spectra of specific wavelengths are selected, and relevant information is lost. The proposed classification scheme has been programmed and tested. With it, the average computation time for the classification of any new measured spectrum has been estimated to be 0.0107 ± 0.0013 s.

## 4. Materials and Methods

### 4.1. Animal Model

Twenty-two male C57BL/6 mice of 16 weeks of age and a weight of 25 ± 5 g were obtained from the Vivarium at the Experimental Medicine Unit of the Hospital General de Mexico, Mexico City, Mexico and were kept under controlled conditions. Both food and water were allowed ad-libitum. The whole procedure was approved by the Institutional Ethics Review Board at the “Hospital General de Mexico” (approval number: DI/16/UME/05/048).

To induce different stages of steatosis, the mice were randomized to be fed either MCC (MP Biomedicals, CA, USA) or MCD diet for a period of two and eight weeks as described below. Animals whose NAFLD evolved to advanced stages as diagnosed by means of histological evaluation after the slaughter were excluded from the study to avoid the complexities of the spectral analysis inherent to the disease.

### 4.2. Liver Sample Collection

After disease induction time (two or eight weeks), animals were anesthetized with Xylazine-Ketamine and euthanized. Liver samples from the right lobe were collected and preserved in sterile cold phosphate buffered saline PBS for immediate EFS assessment as described below. The left lobe was divided to either be embedded in Tissue-tek OCT (Sakura Finetek, Torrance, CA, USA) and stored at −20 °C until assayed or be fixed in 3.7% formaldehyde-PBS solution to be embedded in paraffin.

### 4.3. Hepatic Fat Content Assesement

Histological evaluation of the degree of NAFLD was performed according to the non-alcoholic fatty liver disease activity score (NAS) [32] in Hematoxylin-Eosin tainted sections. Contents of lipids in liver were evaluated in frozen sections tainted with Oil-Red O (Abcam, Cambridge, MA, USA) and computed by a morphometric analysis using Image J software (NIH, Bethesda, MD, USA).

### 4.4. Instrumentation and Fluorescence Spectroscopic Measurements

The fluorescence instrumentation used in this study has been reported elsewhere [19,33]. Briefly, as excitation light sources, LEDs operating at 330 nm (UVTOP330, SETi, Columbia, SC, USA), 365 nm (NCSU033A(T), Nichia, Anan, Japan), 385 nm (380-1WUE, Violed Int., Taichung, Taiwan), 405 nm (405-1WUE, Violed Int., Taichung, Taiwan) and 415 nm (410-1WUE, Violed Int., Taichung, Taiwan) at 0.125, 1.25, 1.35, 0.55, and 0.65 mW optical powers, respectively, were used. A QE65000-ABS spectrometer (Ocean Optics Corp., Winter Park, FL, USA) which operates in the region from 200 to 1100 nm was used. The fiber optic bundle (QR400-UV-VIS, Ocean Optics Corp., Winter Park, FL, USA) has two branches, one with a single fiber optic to collect the light and deliver it to the spectrometer, and the other with a circular array of six fibers that are employed for irradiation. All fibers have a diameter of 400 µm. The operation of both spectrometer and light source runs through a custom programmed LabVIEW interface. A diagram of the instrumentation used is shown in Reference [19].

Ex-vivo endogenous fluorescence spectra were acquired from a total of 22 liver tissue samples collected from the same number or subjects: nine for the MCC group, six for the MCD2w group and seven for the MCD8w group for all excitation lights used, except for the excitation light of 415 nm for which the MCD2w group consisted of only three subjects. Measurements were carried out at the same time as the previously reported diffuse reflectance measurements [19]. The developed instrumentation and methodology enabled us to record the spectroscopic measurements in one liver with each excitation wavelength in a maximum of one minute. Total time for all measurements, including diffuse reflectance and all the fluorescence spectra of each subject was around six minutes; that includes taking measurements in several points on the liver. Measurements were taken in a darkened room placing the probe tip in close contact with the liver surface at different points through the Glisson’s capsule without puncturing the liver at a spatial density of 22 points per square centimeter. This process was executed each time by the same operator to ensure that a similar constant pressure was always applied. Before the spectral measurements of liver samples, a dark spectrum was collected by blocking the light input to the spectrometer after a 15 min warm-up interval. The dark spectrum was subtracted from each measured spectrum; also, a boxcar smoothing filter included in the acquisition software was activated to eliminate the high frequency noise produced by the spectrometer. The integration time of the spectrometer was fixed to 100 ms. The spectrometer setting was such that its resolution is equal to 0.77 nm, which yields typically more than 300 measuring points in each spectrum. As spectra were limited to a suitable range in dependency of the excitation light, measuring points resulted in the following manner reported as: excitation wavelength (measurement interval) number of points in the observed fluorescence spectrum 330 nm (435.5–721.2 nm) 372 points, 365 nm (435.5–722 nm) 373 points, 385 nm (440.9–741.6 nm) 392 points, 405 nm (490.9–795.8 nm) 400 points, and 415 nm (500.2–750.7 nm) 328 points.

### 4.5. Statistical Analysis

Measured spectra were corrected to take into account differences in the sensitivity of the spectrometer at different wavelengths using a calibration lamp (LS-1-CAL-INT) in a suitable setup with a custom LabVIEW routine. Afterward, all spectra were normalized to an arbitrary intensity value of one at a wavelength of 600 nm to eliminate the differences related to the irradiation geometry while maintaining the spectral distribution of the collected light.

A total of 1870 fluorescence spectra were acquired—683 spectra for MCC group, 583 for MCD2w group, and 604 for MCD8w group. Their distribution according to the excitation light is shown in Table 5. For each group, the mean of all the measured spectra at one wavelength as well as standard deviation were calculated.

Afterward, the area under the curve (AUC) of the spectra at each wavelength was calculated. This parameter was used in two different ways—to evaluate its use as a classification parameter to differentiate between groups, and afterward, to correlate the fluorescence spectra with the liver lipid content. After these tests, a more rigorous and sophisticated multi-variate statistical analysis was developed. An orthogonal transformation based on principal component analysis (PCA) was performed over the complete spectral data set to reduce the number of variables while losing minimum information [34]. Principal components (PCs) that explained most of the variance (>90%) and that correspond to the eigenvalues before the cut-off point or “elbow” of the “scree” plot [35] were selected for the creation of a classification model. Next, a supervised multivariate classification scheme was applied using a Discriminant Analysis (DA) model, where the independent variables were the previously selected PCs, and considering the membership to the groups MCC, MCD2w, and MCD8w as the categorical dependent variables. For this discriminant model, a linear variant is sometimes preferable, especially when there is a limited number of available observations due to its better stability to variations in the statistical data [36], but if the data set fails the test of the assumption of equality of variance-covariance within groups then the quadratic variation is recommended. That was the case in this work, thus the quadratic discriminant analysis (QDA) was used. For classification purposes, all observations were used as training data. The confusion matrix for this classification scheme was then constructed and the classification rate errors were calculated in as the value in percent of misclassifications relative to the actual number of observations.

Also, a leave-one-out cross-validation scheme was carried out using all but one of the observations to train a new classification model every time. That model was used to predict which group the excluded observation should be assigned to and then verifying whether the classification was correct or not. The procedure was repeated for each observation so that each one of the observations could be classified by a model of the other observations. Afterward, the validation confusion matrix and the cross-validation rate errors were determined in a way analogous to the previous classification strategy.

A canonical score plot of DA was implemented to find new coordinate axes that offer a visualization of maximum-separation among the groups. In this case, there will be only two canonical variables, since the classification consists of only three groups or classes. A grid is implemented in the space of the canonical variables, and a supervised classification of each point of the grid is performed to assign the belonging of each point to one of the evaluated classes. In this way, the decision boundaries for QDA were determined and plotted in the corresponding canonical score plot.

Probability estimates obtained from the above classification model were then used as scores to obtain the receiver operating characteristic (ROC) curves fixing in every case one state as positive (i.e., belonging to its class) and the other two as negative. The ROC curve plots the proportion of true positives (sensitivity) against the proportion of false positives (1 − specificity) for every possible value of the test as threshold (in our case the posterior probability obtained from the classification model) [37]. The area under the curve (AUC) was obtained for every ROC curve and they were used for comparison of the discriminative power of the different tests. Optimal values of sensitivity and specificity for each ROC curve were found using the criterion of the Youden index which is calculated as the cut-point where the maximum of (sensitivity + specificity − 1) occurs [38]. The maximum value of the Youden statistic corresponds to the optimum cut-off point of the ROC curve that optimizes the predictors differentiating ability when equal weight is given to sensitivity and specificity. The software MATLAB R2018b (Mathworks, Natick, MA, USA) was used to perform the statistical analysis.

## 5. Conclusions

We have shown in an animal model that endogenous fluorescence spectroscopy (EFS) excited with a LED-based source can be a valuable tool for the evaluation of steatosis in its early stages, without the necessity of puncturing the liver. It was shown that the systemic deposition of fat in the liver causes distinctive patterns of endogenous fluorescence emission. Using the area under the curve of fluorescence spectra as indicator, it was possible, with several excitation wavelengths, to distinguish between MCD2w and MCD8w groups, even when histopathological morphometric quantification fails. The high correlation between fluorescence measurements and the fat histopathological quantification points to the possibility of using EFS for liver fat content gradation. Moreover, the distinctive fluorescence band around 620 nm could be used as an optical marker for a simple and fast local estimation of the steatosis degree in the liver. The results of the multivariate statistical analysis demonstrate that it is possible to use EFS measurements to correctly categorize the different grades of steatosis in its early stages. Measurements using the 330 nm excitation light exhibited relative worse sensibility and specificity, while for the others, excitation lights results were better; nevertheless, with the 405 nm excitation, the classification was nearly perfect. As EFS is characterized by its low cost, portable instrumentation, diminished invasiveness, promptness, and the possibility of reducing the human error during tissue evaluation, it could offer great advantages for liver graft assessment, especially from living donors.

## Figures and Tables

**Table 1:** Correlation between fluorescence measurements and lipid quantification from morphometry.

Correlation Test		AUC_330 nm_ vs. Lipid Cont. ^a^	AUC_365 nm_ vs. Lipid Cont. ^a^	AUC_385 nm_ vs. Lipid Cont. ^a^	AUC_405 nm_ vs. Lipid Cont. ^a^	AUC_415 nm_ vs. Lipid Cont. ^a^
Spearman	Correl. coeff. ^b^	0.57538 *	0.24901	0.7572 *	0.75833 *	0.72632 *
	*p*-value	0.00508	0.26378	4.50604 × 10^−5^	4.32295 × 10^−5^	4.29233 × 10^−4^

^a^ Lipid content. ^b^ Correlation coefficient. * Positive correlation at *p <* 0.05 significance level.

**Table 2:** Confusion matrix of the quadratic discriminant analysis (QDA) classification and cross validation models performed on the first six principal components of spectral data gathered with different excitation lights on the three possible liver groups—MCC, MCD2w, and MCD8w.

Excitation Wavelength (nm)	Actual Group (A-Number ^a^)	Predicted Group (Classification, Cross-Validation ^b^)			Percent of Success ^c^
		MCC	MCD2w	MCD8w	
330	MCC (132)	117, 115	15, 16	0, 1	88.6, 87.1
	MCD2w (130)	2, 4	128, 126	0, 0	98.5, 96.9
	MCD8w (111)	0, 1	5, 5	106, 105	95.5, 94.6
	Total (373)	119, 120	148, 147	106, 106	
365	MCC (133)	133, 133	0, 0	0, 0	100.0, 100.0
	MCD2w (125)	4, 6	121, 118	0, 1	96.8, 94.4
	MCD8w (116)	0, 0	0, 1	116, 114	100.0, 98.3
	Total (374)	137, 139	121, 119	116, 115	
385	MCC (136)	135, 135	1, 1	0, 0	99.3, 99.3
	MCD2w (129)	1, 4	127, 124	1, 1	98.5, 96.1
	MCD8w (128)	1, 1	0, 0	127, 126	99.2, 98.4
	Total (393)	137, 140	128, 125	128, 127	
405	MCC (145)	145, 145	0, 0	0, 0	100.0, 100.0
	MCD2w (131)	2, 3	129, 128	0, 0	98.5, 97.7
	MCD8w (125)	1, 1	1, 1	123, 123	98.4, 98.4
	Total (401)	148, 149	130, 129	123, 123	
415	MCC (137)	137, 136	0, 0	0, 1	100.0, 99.3
	MCD2w (68)	1, 1	67, 67	0, 0	98.5, 98.5
	MCD8w (124)	2, 2	0, 0	122, 123	98.4, 99.2
	Total (329)	140, 139	67, 67	122, 124	

^a^ Actual number of observations, corresponding to 100%, according to the supplied diet. ^b^ Number of observations assigned to the given group by (classification model, cross validation). ^c^ Percentage of successful appointments of the observations to their actual group by (classification, cross-validation).

**Table 3:** Classification error rate of the quadratic discriminant model applied using the classification and cross-validation strategies.

Excitation Light (nm)	MCC (Classif., Cross)% ^a^	MCD2w (Classif., Cross)% ^a^	MCD8w (Classif., Cross)% ^a^	Total (Classif., Cross)% ^a^
330	11.4, 12.9	1.5, 3.1	4.5, 5.4	5.9, 7.2
365	0.0, 0.0	3.2, 5.6	0.0, 1.7	1.1, 2.4
385	0.7, 0.7	1.6, 3.9	0.8, 1.6	1.0, 2.0
405	0.0, 0.0	1.5, 2.3	1.6, 1.6	1.0, 1.3
415	0.0, 0.7	1.5, 1.5	1.6, 1.6	0.9, 1.2

^a^ Error rate, in percent, expressed for (classification, cross-validation) schemes.

**Table 4:** Area under the curve (AUC) for the ROC curves, together with the indexes that asses the performance of fluorescence spectroscopy as a diagnostic tool of liver-steatosis degree, obtained from the spectra recorded under each excitation wavelength in every of the three classification groups.

Excitation Wavelength (nm)	Parameter	MCC ^a^	MCD2w ^b^	MCD8w ^c^	Average
330	AUC	0.980	0.973	0.995	0.983
	Optimal Sensitivity	0.909	0.985	0.973	0.956
	1 − Optimal Specificity	0.041	0.128	0.031	0.067
	Optimal cut-off	0.359	0.341	0.287	
365	AUC	0.998	0.990	0.998	0.995
	Optimal Sensitivity	0.977	0.96	1.000	0.979
	1 − Optimal Specificity	0.008	0.032	0.008	0.016
	Optimal cut-off	0.841	0.295	0.116	
385	AUC	0.994	0.991	0.992	0.993
	Optimal Sensitivity	0.985	0.977	0.977	0.980
	1 − Optimal Specificity	0.016	0.049	0.011	0.025
	Optimal cut-off	0.847	0.155	0.434	
405	AUC	0.997	0.997	0.993	0.996
	Optimal Sensitivity	0.986	0.992	0.992	0.990
	1 − Optimal Specificity	0.004	0.007	0.007	0.006
	Optimal cut-off	0.548	0.554	0.222	
415	AUC	0.994	0.996	0.997	0.996
	Optimal Sensitivity	0.985	0.985	0.992	0.988
	1 − Optimal Specificity	0.031	0.027	0	0.019
	Optimal cut-off	0.456	0.363	0.003	

**^a^** MCC vs (MCD2w + MCD8w); **^b^** MCD2w vs (MCC + MCD8w); **^c^** MCD8w vs (MCC + MCD2w).

**Table 5:** Distribution of the number of measured fluorescence spectra per group and excitation wavelength.

	330 nm	365 nm	385 nm	405 nm	415 nm	Total
MCC	132	133	136	145	137	683
MCD2w	130	125	129	131	68	583
MCD8w	111	116	128	125	124	604
Total	373	374	393	401	329	1870
